# A Systematic Review on Biomarkers: Are There Reliable Molecular Biomarkers in Patients With Rheumatoid Arthritis-Associated Interstitial Lung Disease?

**DOI:** 10.7759/cureus.66422

**Published:** 2024-08-08

**Authors:** Sheezara T Lira, Maxsuel R Costa, Wérgila R Gonçalves Barros, Jucier Gonçalves Junior

**Affiliations:** 1 Internal Medicine, Universidade Federal do Cariri (UFCA), Barbalha, BRA; 2 Internal Medicine, Escola de Saúde Pública (ESP), Juazeiro do Norte, BRB; 3 Rheumatology, Universidade Federal do Cariri (UFCA), Barbalha, BRA

**Keywords:** : rheumatoid arthritis, disease pathogenesis, s: epidemiology, interstitial lung disease in rheumatoid arthritis, s: biomarkers

## Abstract

Despite advances in the study of rheumatoid arthritis-associated interstitial lung disease (RA-ILD), the pulmonary manifestation remains an important cause of morbidity and mortality. However, there is a lack of biochemical markers for this manifestation in the literature. Therefore, the objective of this study was to carry out a qualitative systematic review on biochemical markers associated with RA-ILD in the PubMed, Web of Science, Embase, Cochrane Library, and Virtual Health Library (VHL) between January 2015 and July 2024, using the following descriptors: #1 "biomarkers" (MeSH) AND #2 "rheumatoid arthritis" (MeSH) AND #3 "Lung Diseases, Interstitial" (MeSH). Of the 1497 articles found, 27 presented eligibility criteria. The findings were divided into three sessions: "Main biomarkers for RA-ILD," "Other biomarkers for RA-ILD activity," and "Other biomarkers for RA-ILD prognosis." Among the evaluated markers, KL-6, RF, ACPA, ESR, and CRP appear to have prognostic value and association with damage in patients with RA-ILD. The association of some molecules such as sPD-1, sCD25, VCAM-1, MCP-1, and ADMA with tissue damage is intriguing. Longitudinal and randomized studies are imperative to comprehensively delineate the history of RA-ILD and evaluate potential serum biomarkers.

## Introduction and background

Rheumatoid arthritis (RA) is a chronic, multisystem, idiopathic disease characterized by a Th1-type immune response targeting the synovial membrane [1]. Its incidence is estimated to affect approximately 1% of the world population, with interstitial lung disease (ILD) representing a significant source of morbidity and mortality among its extra-articular manifestations [2-4].

Previous studies have shown that mortality rates in patients with RA-associated ILD (RA-ILD) can be 1.27-3 times higher, especially within the first few years of disease onset, compared to those without ILD [3,5]. Certain radiological patterns, such as usual interstitial pneumonia (UIP), have been identified as more aggressive and predictive of a poorer prognosis [6]. Furthermore, the clinical course of RA-ILD can be challenging and variable [7,8]. Besides that, in RA-ILD, Th2-type immune response may have a major role.

While markers like the rheumatoid factor (RF) and anti-citrullinated peptide antibody (ACPA) are known to correlate with disease activity in RA [9,10], there remains a gap in our understanding despite advancements in biochemical markers for RA-ILD [1,11]. Specifically, there is a lack of substantial theoretical contributions and specific biochemical markers tailored for RA-ILD screening within at-risk groups and guidance on treatment decisions [12].

Hence, the objective of this study was to carry out a qualitative systematic review of the literature on biochemical markers associated with RA-ILD based on the following question: What practical contributions does current scientific literature offer regarding the association between biochemical markers and RA-ILD? Our hypothesis is that given that RA's pathophysiology is characterized by a significant Th1-type immune response, but in RA-ILD there is a greater Th2-type immune response, serum biochemical markers may predict disease activity and correlate positively with disease prognosis in pulmonary tissue.

## Review

Materials and methods

Literature Review

A qualitative systematic review of the literature was conducted following the Preferred Reporting Items for Systematic Reviews and Meta-Analyses (PRISMA) protocol. Electronic databases, including PubMed, Web of Science, Embase, Cochrane Library, and Virtual Health Library (VHL), were searched using the following strategy: #1 "biomarkers" (MeSH) AND #2 "rheumatoid arthritis" (MeSH) AND #3 "Lung Diseases, Interstitial" (MeSH) from January 2015 to July 2024. The year 2015 was chosen as the starting point due to the publication of the disease description.

The study followed the PICOS acronym, where "P" represents patients with RA-ILD, "I" denotes the clinical-epidemiological characterization of the pulmonary manifestations of RA-ILD, "C" signifies the healthy control group, and "O" indicates the outcomes of the pulmonary manifestations of RA-ILD.

Data Collection

Data collection occurred between December 2023 and July 2024. Articles were initially screened based on their titles and abstracts. Two researchers independently collected data, with a third senior researcher resolving any discrepancies or uncertainties. Following this initial selection, each article was thoroughly read, and its most relevant findings were compiled in a table, including authors and year of publication, country, qualitative assessment, study type, age (years), number of female participants (n), clinical manifestations of RA-ILD, tomographic patterns, spirometric changes, smoking status, ACPA positivity, C-reactive protein (CRP), erythrocyte sedimentation rate (ESR), and other positive biomarkers present in the sample.

To assess the quality of each study, the Study Quality Assessment tools provided by the National Heart, Lung, and Blood Institute (NHLBI) were used [13]. This tool is a compilation of several tools for analyzing each type of study. For cohorts, we used the "Quality Assessment Tool for Observational Cohort and Cross-Sectional Studies," while case-control studies were assessed by means of the "Quality Assessment of Case-Control Studies." These tools categorize studies as "good," "fair," or "poor" based on the presence or absence of key methodological elements corresponding to each study type [13].

Eligibility Criteria

**Figure 1:**
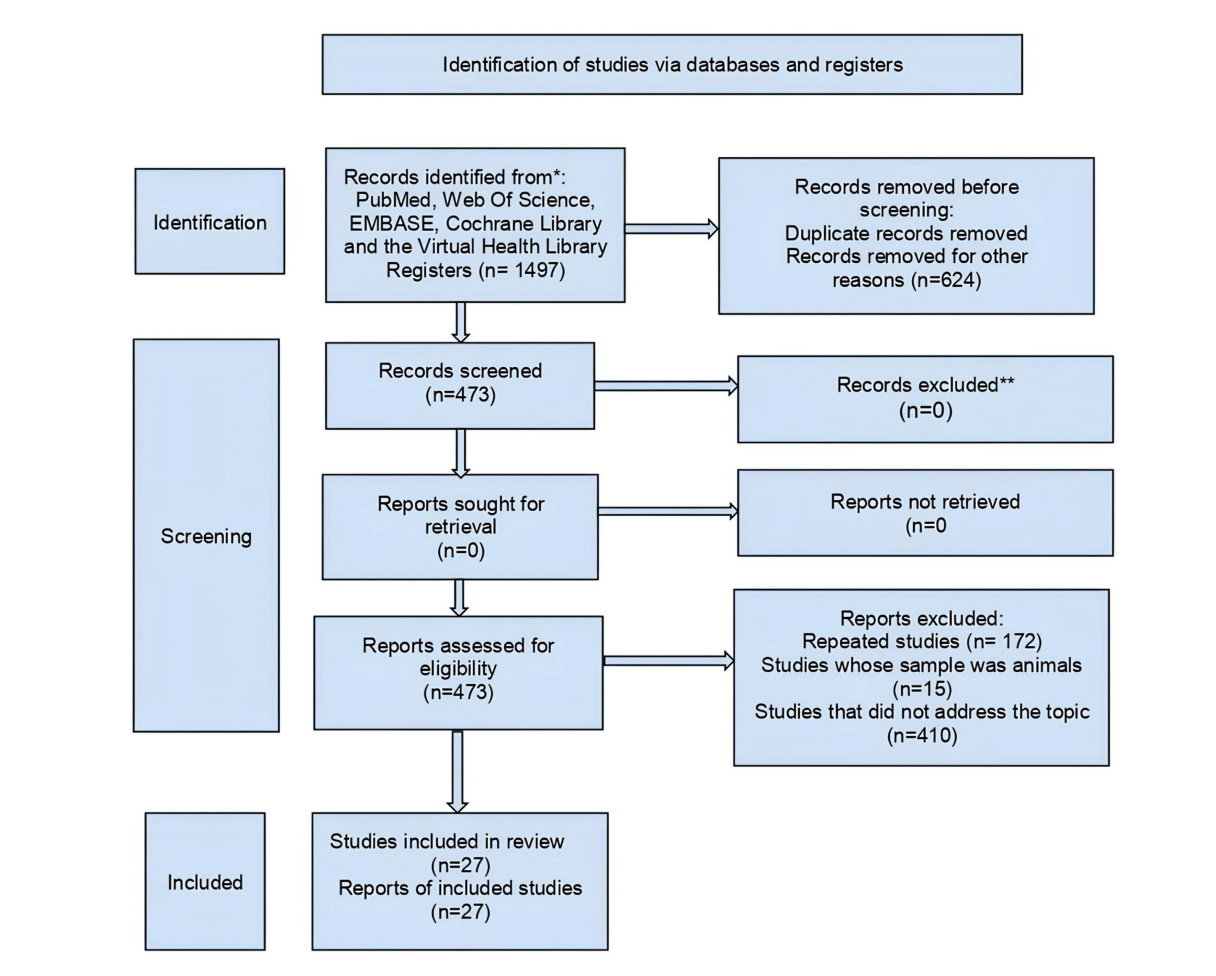
PRISMA flow diagram PRISMA: Preferred Reporting Items for Systematic Reviews and Meta-Analyses

Articles written in Portuguese, English, and Spanish were chosen if they were original, complete, and relevant to the study topic. Inclusion criteria prioritized suitability for the review's objectives, availability and transparency of data, and methodological rigor particularly in clinical, comparative, and observational studies. Exclusion criteria encompassed review or experimental articles, studies involving animal models, brief comments, editorials, communications, and letters to the editor (Figure 1).

Ethical Issue

Considering this is a systematic literature review, Resolution 510/16 of the Brazilian National Health Council (CNS, acronym in Portuguese) exempts the requirement for approval by a human research ethics committee. This review study has been registered on the PROSPERO platform under registration number CRD42024564385.

Results

According to the research strategy, 1497 articles were identified, from which 27 met the eligibility criteria. Table 1 presents the results of applying the eligibility criteria.

**Table 1:** Main findings ARG1: arginase 1; ACPA: anti-citrullinated peptide antibody; anti-CEP-1: anti-citrullinated alpha enolase peptide-1; ADMA: asymmetric dimethylarginine; BIRC5: baculoviral inhibitor of apoptosis repeat containing 5; CA: carbohydrate antigen; CEA: carcinoembryonic antigen; CLEC12A: C-type lectin domain family 12 member A; CRP: C-reactive protein; deciliter: dL; DLCO: diffusing lung capacity for carbon monoxide; ET-1: endothelin-1; EPC: endothelial progenitor cells; ESR: erythrocyte sedimentation rate; FEV1: forced expiratory volume in the first second; FVC: forced vital capacity; HE4: human epididymis protein-4; HLA: human leukocyte antigen; h: hour; ILD: interstitial lung disease; IL-13: interleukin-13; IP-10/CXCL10: interferon-inducible protein 10; KL-6: Krebs von den Lungen-6; LINC02967: long intergenic non-protein coding RNA; LOXL2: lysyl oxidase like-2; mg: milligram; mm: millimeter; MMP-7: matrix metalloproteinase-7; MMP-13: matrix metalloproteinase-13; MCP-1: monocyte chemoattractant protein-1; MKI67: Ki-67 proliferation marker; MS4A4A: membrane spanning 4 A4A domains; NSIP: non-specific interstitial pneumonia; OLFM4: olfactomedin 4; RF: rheumatoid factor; SD: standard deviation; SORT1: sortilin 1; sPD-1: soluble programmed death molecule; UIP: usual interstitial pneumonia; VCAM-1: vascular cell adhesion molecule-1; sCD25: soluble interleukin-2 receptor alpha chain; TYMS: thymidylate synthetase *Analyzed by the Quality Assessment Tool for Observational Cohort and Cross-Sectional Studies **Analyzed by the Quality Assessment of Case-Control Studies

Author (year)	Country	Study type	Quality assessment	Age (years)	Female sex (n)	Clinical manifestations of RA-ILD (n, %)	Tomographic patterns (n, %)	Spirometric changes (mean±SD)	Smoking (n, %)	RF+ (UI/mL, mean±SD)	ACPA+ (UI/mL, mean±SD)	CRP (mg/dL, mean±SD)	ESR (mm/h, mean±SD)	Other positive biomarkers in the sample
Pulito-Cueto et al. (2022) [14]	Spain	Cohort prospective*	Good	66.5±10.1	9	-	UIP (11, 52.4%); probable UIP (2, 9.5%); NSIP (7, 33.3%); non-NSIP (1, 4.8%)	FVC (95.2±24.1); FEV1 (92.2±21.0); FEV1/FVC (77.8±9.1); DLCO (43.3±15.9 mL/min/mmHg/L)	13/21, 65%	-	-	1.1±1.1	22.8±27.2	VCAM-1, MCP-1, ADMA
Pulito-Cueto et al. (2023) [15]	Spain	Cohort prospective*	Good	61.1±9.2	43	-	UIP (33/43, 39.8%); NSIP (34/43, 41%); probable UIP (10/43, 12%); indeterminate for UIP pattern (2/43, 2.4%); non-NSIP (4/43, 4.8%)	FVC (80.4±25.7%); FEV1 (79.1±24.9%); DLCO (39.6±17.6 mL/min/mmHg/L)	60/91, 65.9%	-	-	-	-	ET-1
Chen et al. (2015) [16]	China	Cohort retrospective*	Good	53.0±14.2	29	Cough (5/12%); dyspnea (8/20%)	-	FEV1- (79.3%±15.9%); FVC (76.76±13.4%); DLCO (73.3%±22.0 mL/min/mmHg/L)	5/41, 12%	795.0±1966.3	255.2±187.0	-	-	MMP-7, IP-10/CXCL10
Doyle et al. (2015) [17]	United States	Cohort retrospective*	Good	66.0±10.0	36	-	-	FEV1 (69%); FVC (70%); DLCO (57 mL/min/mmHg/L)	29/48, 63%	275.0±448.0	189.0±137.0	-	-	MMP-7
Qin et al. (2022) [18]	Mexico	Cohort retrospective*	Good	62.8±8.7	45	-	-	-	16/21, 33%	-	-	26.0±20.0	57.0±25.0	KL-6
Chen et al. (2022) [19]	China	Cohort prospective*	Fair	63.0±7.7	30	Cough (4/38, 10.5%); dyspnea (4/38, 10.5%)	-	FEV1 (79-86%); FVC (73-86%); DLCO (59-94 mL/min/mmHg/L)	5/38, 13.15%	473.83±799.0	374.4±125.6	-	-	CXCL11, MMP-13
Alunno et al. (2018) [20]	Italy	Cohort prospective*	Good	61.7±0.8	196	Rheumatoid nodules (47/196, 23%); erosions (153/196, 63%); vasculitis (6/196, 3%); peripheral neuropathy (1/196, 1%)	-	-	-	-	-	-	-	Anti-CEP-1
Wang et al. (2016) [21]	China	Cohort retrospective*	Fair	63.4±11.3	12	-	-	Airflow limitation (FEV1/FVC <70%)	6/28, 21.4%	520.5±40.0	296.4±203.0	-	-	CA15-3, CA125, CA19-9
Sargin et al. (2018) [22]	Turkey	Case-control**	Good	60.1±11.5	30	-	-	-	5/43, 11.62%	35.9	19.05	21.04	70	CA15-3, CA125
Yu et al. (2023) [23]	China	Case-control**	Good	68.0±6.0	39	-	-	-	-	-	-	-	-	KL-6
Avouac et al. (2020) [24]	France	Cohort prospective*	Good	71±15	22	-	-	FVC (5-50%); DLCO (28-138 mL/min/mmHg/L)	24/40, 60%	-	-	-	-	KL-6
Wang et al. (2022) [25]	China	Case-control**	Good	54.8±3.1	134	Cough (55/162, 33.9%); dyspnea (34/162, 20.9%); lung congestion (28/162, 17.3%); dry skin (38/162, 23.5%); cyanosis (3/162, 1.85%)	-	-	51/162, 31.5%	235.3±182.8	467.8±375.9	63.0±20.6	50.3±8.5	Uric acid
Fu et al. (2018) [26]	China	Case-control**	Good	64.9±12.0	30	-	UIP (10.5/30, 35%); non-UIP (28/30, 93.3%)	FVC (5-78, 81%); DLCO (28-48, 52 mL/min/mmHg/L)	-	140.5	394.5±877.0	1.4±3.6	38.5±26.2	LOXL2
Just et al. (2017) [27]	Denmark	Cross-sectional*	Fair	70.0±15.0	6	-	-	FEV1 (39-86%); DLCO (46-73 mL/min/mmHg/L)	2/10, 20%	-	-	-	-	Circulating fibrocytes
Chen et al. (2019) [28]	China	Case-control**	Good	60.9±10.3	79	-	-	-	-	-	-	49.3±23.4	-	Platelet/lymphocyte ratio, lymphocyte/monocyte ratio, neutrophil/lymphocyte ratio
Xu et al. (2021) [29]	China	Case-control**	Good	65.7±9.4	36	Dry cough (21, 61.8%); chest tightness after exercise (14, 41.2%); shortness of breath (6, 17.6%)	-	-	15/58, 25.9%	-	-	32.8±44.3	54.2±34.3	sPD-1
Pulito-Cueto et al. (2020) [30]	Spain	Case-control**	Good	66.8±10.2	9	-	UIP (11/20, 55%); probable UIP (1/20, 5%); NSIP (7/20, 35%); non-NSIP (1/9, 5%)	FEV1 (92.2±21.0%); FEV1/FVC (77.8±9.1%); DLCO (43.3±15.9 mL/min/mmHg/L)	13/20, 65%	-	-	1.1±1.1	22.8±27.2	EPC
Liang et al. (2021) [31]	China	Cohort retrospective*	Good	62.0±8.0	70	-	-	-	2/70, 2.9%	-	-	13.5±18.0	39.0±42.0	HE4
Tanaka et al. (2021) [32]	Japan	Cohort retrospective*	Good	73.0±4.0	22	Dyspnea (22/22, 100%)	-	-	20/33, 51.3%	-	-	-	-	KL-6
Del Angel-Pablo et al. (2020) [33]	Mexico	Case-control**	Good	52.0±26.0 (81.5%)	53	-	NSIP (47/65, 73.3%); UIP (10.8/65, 16.7%); cryptogenic organizing pneumonia (3.25/65, 5%); lymphocytic interstitial pneumonia (2.16/65, 3.33%)	FEV1 (18-145%); FVC (26-147%)	22/65, 37.93	-	-	1.9	35.5	Anti-HLA
Wu et al. (2020) [34]	China	Cross-sectional*	Fair	65.7±9.4	36	Cough (20/30, 66.7%); dyspnea (7/30, 23.3%); chest congestion (13/30, 43.3%)	-	FVC (79.8±20.1%); DLCO (54.2±15.0 mL/min/mmHg/L)	15/58, 15%	-	-	54.2±34.3	32.8±44.3	sPD-1
Kronzer et al. (2023) [35]	United States	Case-control**	Fair	67.0±10.0	65	-	-	-	8/84, 10%	-	-	3.0±6.0	-	ACPA
Paulin et al. (2022) [36]	Argentina	Cohort prospective*	Fair	63.04±2.04	28	Dyspnea (65/65, 100%)	UIP (16/47, 34%); probable UIP (11/47, 23%)	FVC (76.76%); DLCO (57 mL/min/mmHg/L)	31/47, 65.9%	-	-	-	-	IL-13
Lin et al. (2022) [37]	China	Case-control**	Fair	63.4±8.2	31	Dyspnea (18/27, 66.7%)		FEV1 (92.4±20.6%); FVC (86.3±18.9%); DLCO (72.9±22.2 mL/min/mmHg/L)	12/46, 26.1%	-	-	55.6±108.9	49.1±24.8	HE4
Moon et. al. (2021) [12]	South Korea	Cohort prospective*	Fair	66.4±8.2	107	-	-	FEV1 (92.3±21.5%); FVC (85.6±16.6%); DLCO (71.9±19.5 mL/min/mmHg/L)	39/153, 25.5%	244.2±500.5	199.6±166.9	-	-	MMP-7, sPD-1, KL-6
Cao et al. (2024) [38]	China	Cohort prospective*	Good	59.9±15.5	72	Swollen joints (5.03/72±6.7); rheumatoid nodules (7/72, 9.7%); ILD (15/72, 20.8%); renal involvements (7/72, 9.7%); hematological involvements (13/72, 18.1%); metabolic disorders (37/72, 51.4%)	-	-	-	444.4±827.7	188.4±103.9	34.3±94.6	42.2±30.1	sCD25
Wierczeiko et al. (2024) [39]	Germany	Case-control**	Fair	-	12	-	UIP (5/12, 41.66%); NSIP (2/12, 16.6%)	-	-	-	-	-	-	ARG1, TYMS, SORT1, MKI67, OLFM4, BIRC5, MS4A4A, CLEC12A, and LINC02967

The majority of studies were cohorts (59.25%) [12-21,24,27,31,32,34,36,38], followed by case-control studies (40.74%) [21,23,25,26,28-30,33,35,37,39] (as seen in Table 1). Among these, approximately 25.9% originated from Europe (Spain, Italy, France, Germany, and Denmark) [14,15,20,24,27,30,39], followed by Asia with 55.5% (with most papers originating from China, while Japan, Turkey, and South Korea were in the minority) [12,16,19,21-23,25,26,28,29,31,32,34,37,38], South America and the Caribbean with 11.1% [18,33,36] (with papers from Argentina and Mexico), and North America (United States) with 7.4% [35,17] (Table 1).

In the quality analysis, most studies (66.6%) were classified as "good," while the rest were rated as "fair" (Table 1). A total of 1,779 patients with RA-ILD were included in the sample, as detailed in Table 1. The majority (70.6%) of the patients were female, with a mean age of 63 years, a mean disease duration of 6.78 years, and moderate disease activity (Disease Activity Score in 28 Joints (DAS-28) of 4.51).

The most reported symptoms were cough, dyspnea, and xerosis cutis. Upon physical examination, signs of pulmonary congestion, cyanosis, digital clubbing, and Velcro rales were among the most frequently observed changes (Table 1).

In tomographic evaluations, the most observed patterns were UIP and non-specific interstitial pneumonia (NSIP); however, there was controversy among studies. Some authors reported the UIP pattern as more prevalent, while others pointed to NSIP [14,15,26]. Additionally, patterns such as cryptogenic organizing pneumonia and lymphocytic interstitial pneumonia were also noted [12,14,34]. Pulmonary function tests indicated a decrease in diffusing lung capacity for carbon monoxide (DLCO) associated with lower levels of forced vital capacity (FVC) as prominent characteristics of RA-ILD (Table 1).

Laboratory findings, as presented in Table 1, revealed an average level of RF of 351.63 U/L, ACPA level of 264.94, CRP level of 23.54 mg/dL, and ESR of 43.35 mm/h. The DAS-28 was considered moderate in most studies.

Several biomarkers have been investigated in the literature. Uric acid measurement has been found to correlate with lung damage findings on high-resolution chest tomography (HRCT), although it lacks specificity [25]. Some biomarkers have shown associations with the severity of NSIP associated with RA-ILD, such as soluble programmed death molecule (sPD-1), which has also been linked to deteriorating lung function [13,24,32,34]. Krebs von den Lungen-6 (KL-6) stands out in predicting severity, demonstrating a reasonable sensitivity and specificity profile, along with an association with worse pulmonary function tests [13,18,24,32]. Circulating fibrocytes studied in a Danish cohort have shown agreement with the severity of pulmonary involvement, disease activity, and worsening of pulmonary function tests [27].

Despite their poor specificity, tumor markers have also been studied as potential biomarkers [18,21,22,31,37]. The main ones related to lung damage include carbohydrate antigen (CA) 19-9, 125, 242, and 15-3, as well as carcinoembryonic antigen (CEA) [18,21,22,31,37]. However, probably due to their low specificity, they did not correlate with worsening lung function or tomographic findings. Human epididymis protein-4 (HE-4) also demonstrated low accuracy, especially for early stages of pulmonary involvement, despite its association with worsening spirometric data and fibrosis findings on HRCT [31,37].

In a study conducted in a Spanish cohort, endothelial progenitor cells (EPC) demonstrated elevated values ​​in pulmonary fibrosis, despite having no relationship with the worsening of lung function [15]. Conversely, vascular cell adhesion molecule-1 (VCAM-1), monocyte chemoattractant protein-1 (MCP-1), and asymmetric dimethylarginine (ADMA) were found to correlate with the severity of RA-ILD, exhibiting good specificity in assessing pulmonary involvement [14,15,30]. Endothelin-1 (ET-1), as investigated by Pulito-Cueto et al., was only related to deteriorating lung function tests [15].

Additionally, anti-human leukocyte antigen (HLA) antibodies were related to HRCT findings of UIP, NSIP, and organizing pneumonia [33]. Other antibodies examined in this study, including anti-citrullinated and native antibodies, with a particular focus on histone-4, histone-2A, and filaggrin, showed potential in predicting the future development of RA-ILD through a scoring system based on their levels, alongside the assessment of risk factors [35].

Matrix metalloproteinase-7 (MMP-7) and interferon-inducible protein 10 (IP-10/CXCL10) were associated with the severity of RA-ILD and demonstrated the ability to identify mild forms of the disease [16,17]. MMP-13 and interferon-gamma inducible protein (CXCL11) showed promise in predicting the prognosis of lung disease [19].

Other markers were studied in this research. Chitinase-3 like-protein-1 (CHI3L1) did not exhibit a favorable sensitivity and specificity profile for identifying lung involvement [23]. Despite their role in the inflammatory response, low cost, and accessibility, the platelet/lymphocyte ratio (PRL), lymphocyte/monocyte ratio (LMR), and neutrophil/lymphocyte ratio (NLR) also have low specificity in predicting lung damage [28].

Interleukin-13 (IL-13) was associated with deteriorating spirometric data, but the study's novelty lies in the potential identification of new anti-IL-13 drugs in the treatment of RA-ILD, given its relation to pathogenesis [36]. Moreover, anti-citrullinated alpha-enolase peptide 1 (anti-CEP-1) demonstrated a possible association with *Porphyromonas gingivalis*, a bacterium studied in severe forms of RA [20].

In a Chinese cohort study, soluble IL-2 receptor α chain (sCD25) was evaluated as a potential biomarker for RA-ILD [38]. Results using the enzyme-linked immunosorbent assay (ELISA) method indicated that it provided good specificity with lung damage and a relationship with HRCT data of UIP and NSIP patterns [38].

Furthermore, a German case-control study assessed several genes [39], revealing nine of them associated with lung involvement in RA: arginase 1 (ARG1), thymidylate synthetase (TYMS), sortilin 1 (SORT1), Ki-67 proliferation marker (MKI67), olfactomedin 4 (OLFM4), baculoviral inhibitor of apoptosis repeat containing 5 (BIRC5), membrane spanning 4 A4A domains (MS4A4A), C-type lectin domain family 12 member A (CLEC12A), and the long intergenic non-protein coding RNA (LINC02967). Among these, ARG1 demonstrated the strongest association with RA-ILD [39].

Lastly, lysyl oxidase like-2 (LOXL2) showed promise in identifying early stages of RA-ILD, and its elevated levels were correlated with worsening pulmonary function tests, indicating its potential as a biomarker [25].

The findings were then divided into "Main biomarkers for RA-ILD," "Other biomarkers for RA-ILD activity," and "Other biomarkers for RA-ILD prognosis."

Discussion

Main Biomarkers for RA-ILD

Higher levels of RF, ACPA, CRP, and ESR were observed in patients with RA-ILD [22,33]; however, there is disagreement among authors. Xu et al. consider elevated ACPA as a risk factor for RA-ILD [29], but other studies [22,33] did not find this association and did not identify high CRP, ESR, and RF values ​​as possible risk factors for RA-ILD. This aligns with the idea that RA-ILD can develop independently of joint disease activity, reinforcing the need for specific markers for pulmonary involvement. It is important to highlight the retrospective [20,22], single-center [19], and small sample size [19,22,33] of the studies analyzed.

Among the new biomarkers associated with RA-ILD, uric acid measurement stands out [25]. The hypothesis is that uric acid plays a role in the pulmonary inflammatory process due to the Th2 response. High values of uric acid ​​(in both serum and bronchoalveolar lavage) were positively associated with UIP patterns on HRCT in RA-ILD, indicating severity. Despite being a cheap and sensitive test, uric acid is not specific, particularly due to its association with cardiovascular diseases and pulmonary hypertension. Additionally, the present study's lack of cardiovascular mapping and relatively small sample size are limitations [25]. However, the small sample size and the failure to remove confounding factors such as patients with cardiovascular disease or pulmonary hypertension are biases in this study.

KL-6 is a cytokine expressed on the surface membranes of bronchiolar epithelial cells, particularly on type II pneumocytes that are damaged or regenerating. KL-6 has received special attention for its association with RA-ILD: exacerbation of respiratory symptoms, desaturation, worse performance on the six-minute walk test, and greater pulmonary impairment on HRCT and spirometry [13,18,24,32]. In a Mexican cohort, KL-6 demonstrated a sensitivity of 61.33% and a specificity of 78.21%, with mean values of 373.65 U/mL in RA-ILD [18]. These findings were corroborated by a French study, which observed RA-ILD with lung involvement >15% on HRCT and UIP pattern in patients with high KL-6 levels [24].

A Korean cohort suggested that KL-6 levels correlate with RA-ILD severity, indicating its potential use as a prognostic marker [13]. However, a Japanese cohort [31] found a higher prevalence of exacerbation in patients with RA-ILD and elevated KL-6 levels. Exacerbation was defined as a drop in O2 saturation below 90% during a six-minute walk test, initiation or increase of glucocorticoids, and absence of other conditions justifying pulmonary worsening [32].

Elevated KL-6 levels were also observed in patients with lower FVC and DLCO values [13,18,24,32]. Despite its promise, the use of KL-6 is limited by its association with infections, adenocarcinoma; and the secondary, retrospective nature, small sampling [18,24,32] and absence of a control group [13,24,32] of published papers limit generalizations.

A cohort from Spain evaluated the relationship between VCAM-1, MCP-1, and ADMA and the severity of RA-ILD. These molecules are involved in vasculopathy and the inflammatory response in RA-ILD [14]. The levels of these molecules were measured using a commercial kit and correlated with spirometric data. The area under the curve (AUC) analysis demonstrated good specificity for identifying lung damage, but it was less effective for differentiating more serious conditions, such as fibrosis, and was, therefore, unrelated to severity findings on HRCT [14]. It is understood that they may be useful for detecting early stages of RA-ILD.

Other Biomarkers for RA-ILD Activity

sPD-1 seems involved in the activation of T lymphocytes and, thus, in the pathogenesis of RA-ILD [34]. Elevated sPD-1 levels were associated with greater reductions in FVC and forced expiratory volume in one second (FEV1), as well as more severe lung involvement on HRCT [34,29], even though they have poor sensitivity and specificity. Further studies with larger patient cohorts are necessary to establish a cutoff value that offers greater specificity for diagnosis.

The quantity of circulating fibrocytes measured by flow cytometry is also cited in the literature as being associated with RA-ILD. A Danish study demonstrated that high quantities of these cells in peripheral blood were associated with worse DLCO levels [27], highlighting this measure as a potential biomarker. However, the sensitivity, specificity, and clinical application in RA-ILD (including correlation with severity, imaging, and disease activity) have not yet been thoroughly evaluated, indicating a significant limitation. Furthermore, the need for advanced technology and the associated high costs currently limit its affordability and accessibility.

The role of tumor markers has also been investigated in RA-ILD. Elevated serum levels of CA19-9, CA125, CA242, CA15-3, HE4, and CEA have been observed in RA-ILD [21,22,31,37]. Nevertheless, no studies have established parallels between marker values and clinical-radiological parameters, such as tomographic involvement in RA-ILD [18,21,22,31,37].

In a Chinese cohort, CA125 showed low sensitivity and specificity for RA-ILD [21]. HE4 was detected in bronchoalveolar lavage fluid in RA-ILD patients [30] with a sensitivity of 65.9% and a specificity of 86%, indicating low precision in detecting early stages of the disease. These findings were supported by a Chinese case-control study [6]. Both studies identified an inverse relationship between HE4 levels and DLCO, as well as FVC values, and identified a link with the emergence of pulmonary fibrosis [31,37]. Although this research points to a potential marker for RA-ILD, more studies are needed to find the early stages of lung involvement.

A US cohort investigated anti-HLA antibodies in patients with RA-ILD, correlating their presence with CRP levels, based on their role in hyperacute lung allograft rejection [33]. The patients had various forms of pulmonary involvement, ranging from UIP and NSIP to organizing pneumonia [33]. Nevertheless, no sensitivity profile or relationship with lung function was demonstrated, requiring more detailed studies to validate these antibodies as biomarkers [33].

In addition to probable biomarkers, a cohort study in Italy investigated anti-CEP-1 [20]. Although this study did not provide relevant information on the association of anti-CEP-1 with pulmonary function tests, tomographic findings, or the prognosis of RA-ILD, it did evaluate the role of anti-CEP-1 in the development of RA-ILD [19]. The cohort identified a potential relationship between anti-CEP-1 and *Porphyromonas gingivalis*, a bacterium implicated in the pathogenesis of RA-ILD and erosive RA, which may explain why non-smoking RA patients develop pulmonary involvement [20]. However, further studies are needed to confirm this relationship and to evaluate lung damage.

Soluble interleukin-2 receptor alpha chain (sCD25) was evaluated in a Chinese cohort and demonstrated a strong relationship with the diagnosis of RA-ILD [38]. This biomarker was associated with various types of RA involvement, such as renal, hematological, and metabolic disorders, but showed a significant association only with pulmonary involvement [38]. sCD25 exhibited low sensitivity and reasonable specificity with a cutoff value of 1.172,20 pg/mL, determined by an immunoenzymatic assay [37]. The main tomographic findings were UIP and NSIP patterns [38]. Despite the small sample size and the lack of specific correlation with lung function data, sCD25 has the potential to become a useful serum biomarker for the early diagnosis of RA-ILD pending further detailed studies.

In Germany, a case-control study involving 12 patients aimed to identify genes related to pulmonary involvement in RA [39]. Among these patients, seven had pulmonary involvement: five with a UIP pattern and two with non-specific fibrosing interstitial pneumonia, all with bilateral lung involvement [39]. The identified genes were ARG1, TYMS, SORT1, MKI67, OLFM4, BIRC5, MS4A4A, CLEC12A, and LINC02967 [39]. The study faced several limitations, including the lack of data on respiratory symptoms and lung function, a small sample size, high costs, and delays in obtaining results [39].

The retrospective nature of the studies [31,32,37,38] and the small sample size [6,20,31,33,37,39] are also factors that make extrapolation difficult.

Other Biomarkers for RA-ILD Prognosis

Some studies have analyzed the relationship of biomarkers with the severity of RA-ILD [15,24,30]. The use of EPC was investigated in a Spanish case-control study [30], which identified EPC as being responsible for the formation of new vessels. This study also showed that levels of these cells, measured through flow cytometry, were more increased in patients with RA-ILD, mainly in those with more severe manifestations of the disease, such as higher values in fibrosis compared to the UIP pattern [30]. However, this study did not find a relationship between EPC levels and pulmonary function tests, nor did it demonstrate a sensitivity and specificity profile, and the sample is small [30].

Another Spanish cohort study also investigated ET-1, a molecule associated with vascular damage and fibrotic processes, to evaluate its relationship with RA-ILD and pulmonary fibrosis [15]. The cutoff value that presented the best sensitivity and specificity of this molecule was 0.88 pg/mL. Higher levels of ET-1 were related to lower FVC and DLCO values [15]. However, further studies are needed to confirm the diagnostic utility of this molecule for RA-ILD.

A case-control analysis performed in the United States compared citrullinated and native autoantibodies to predict RA-ILD [35]. Among the prominent antibodies were histone 4, histone 2A, and filaggrin, with the latter two showing the strongest association with RA-ILD. This study also developed a score for predicting future pulmonary involvement in RA, demonstrating high sensitivity and specificity, although the score without biomarkers also had high specificity [35]. The variables used included smoking status, DAS-28-CRP score, current use of glucocorticoids, body mass index (BMI), filaggrin, histone 4, and histone 2A [35]. Limitations of the study included the lack of correlation with tomographic findings and pulmonary function testing.

An immunoenzymatic assay conducted by Chinese and American cohorts evaluated the use of cytokines and chemokines as biomarkers for RA-ILD, with a focus on MMP-7 and IP-10/CXCL10 [16]. This study assessed the predictive value of these markers, with MMP-7 showing better relative performance [16]. Furthermore, it revealed an association between these cytokines and the severity of lung disease, allowing for the assessment of both severe (pulmonary fibrosis) and milder manifestations [16]. MMP-7 levels were further studied by Doyle et al., in a US cohort [17], which also demonstrated good sensitivity and specificity for this marker in RA-ILD [17]. Additionally, higher levels of MMP-7 were associated with a worse prognosis and the identification of subclinical forms of lung disease [17].

A case-control study in China investigated CHI3L1, a chitinase-3-like-protein 1, as a potential biomarker for RA-ILD [23]. CHI3L1, involved in tissue repair, inflammation, and remodeling, was found to be increased in patients with pulmonary involvement, with a sensitivity of approximately 76% and a specificity of around 62% [23]. Although such a study did not correlate CHI3L1 levels with disease severity, it did show higher levels in patients with pulmonary fibrosis compared to the control group. However, the study had limitations, including potential biases, such as not accounting for risk factors for RA-ILD like smoking. Therefore, more detailed studies are needed in order to better clarify the relationship between this potential biomarker and pulmonary involvement.

Another Chinese cohort study evaluated MMP-13 and CXCL11 to assess their relationship with lung involvement in RA patients [19]. High CXCL11 titers were observed in patients with more severe lung damage, suggesting that this cytokine could serve as a prognostic marker for RA-ILD [19]. Radiographically, the main form of involvement was the non-UIP pattern (not usual pneumonia) [19]. A significant limitation of this study was the lack of correlation between CXCL11 and MMP-13 levels with pulmonary function tests, as well as the absence of sensitivity and specificity evaluations.

In the context of evaluating prognosis, other proteins were also used. A prospective cohort from Argentina compared IL-13 values with declining FVC levels, showing a positive relationship between elevated IL-13 levels and worsening pulmonary function tests [36]. However, this study did not establish a relationship between this interleukin and tomographic data, nor did it provide a sensitivity and specificity profile. It proposed the potential use of anti-IL-13 drugs in treating RA-ILD, but further studies are required [36].

Other potential markers that are more accessible and affordable include PRL, LMR, and NLR, as evaluated in a Chinese case-control study [28], which found that they could influence the development and progression of RA, given their significant roles in the inflammatory response [28]. Among these markers, PRL and NLR showed the strongest association with RA-ILD [28]. The AUC was 0.650, with a cutoff value of 144.625 [28]. Although these biomarkers were deemed reliable, they were not specifically linked to lung damage, necessitating further analysis to establish this connection.

LOXL2, a protein strongly expressed in fibrotic lung tissue, was analyzed in a Chinese case-control study to assess its relationship with RA-ILD [26]. This study found higher levels of LOXL2 in patients with RA-ILD of three months' duration or less, suggesting its predictive value [26]. Elevated LOXL2 levels were associated with worsening pulmonary function tests and RF [26]. Despite the small sample size, this study highlighted such protein as a potential biomarker for the early diagnosis of RA-ILD [26].

Limitations

The limitations of this review include (i) the absence of randomized controlled trials (RCTs) evaluating diagnostic/prognostic biomarkers in RA-ILD, making meta-analysis unfeasible; (ii) lack of standardization in marker analysis methods; (iii) no direct comparison of the markers themselves and small samples; (iv) non-standardization of metrics to assess disease activity between studies for comparison; (v) lack of longitudinal studies to evaluate the medium- and long-term behavior of markers in patients with RA-ILD, both ACPA +/- and RF +/-; and (vi) lack of standardization of diagnostic criteria.

## Conclusions

KL-6, RF, ACPA, ESR, and CRP appear to have prognostic value and association with damage in patients with RA-ILD. The association of some molecules such as sPD-1, sCD25, VCAM-1, MCP-1, and ADMA with tissue damage is intriguing. However, the nature of the studies, the isolated data, and the small sample size make generalizations difficult. Thus, longitudinal and randomized studies are crucial to better understand the trajectory of RA-ILD and to evaluate potential serum biomarkers more comprehensively.
